# Distribution of Breast Density in Iranian Women and its Association with Breast Cancer Risk Factors

**DOI:** 10.5812/ircmj.16615

**Published:** 2013-12-05

**Authors:** Nasrin Ahmadinejad, Sajjadeh Movahedinia, Samaneh Movahedinia, Kourosh Holakouie Naieni, Saharnaz Nedjat

**Affiliations:** 1 Advanced Diagnostic and Interventional Radiology Research Center (ADIR), Tehran University of Medical Sciences (TUMS), Tehran, IR Iran; 2 School of Medicine, Tehran University of Medical Sciences (TUMS), Tehran, IR Iran; 3 Epidemiology and Biostatistics Department, School of Public Health, Tehran University of Medical Sciences (TUMS), Tehran, IR Iran

**Keywords:** Breast Neoplasms, Mammography, Mass Screening

## Abstract

**Background:**

Breast cancer is one of the most common cancers and the first-leading cause of cancer deaths among women in the world. Indeed, breast cancer is ranked as the first malignancy among Iranian women. Breast density, defined as the percentage of fibro glandular breast tissue in mammographic images, is one of the known risk factors for breast cancer. According to American college of radiology-Breast Imaging Reporting and Data System (ACR-BIRADS), mammographic density is divided into four categories. Studies have shown that increased breast density is associated with significant increase in breast cancer risk. Therefore, it is assumed that breast density should be associated with other breast cancer risk factors.

**Objectives:**

The aim of this study was to assess the epidemiologic distribution of breast density of the patients in a referral center in Iran, and to evaluate the association of high breast density and breast cancer risk factors and other factors that may possibly affect the mammographic density according to previous studies.

**Patients and Methods:**

In an analytical cross-sectional study, 728 of those who had referred to Imam Khomeini Imaging Center either for diagnostic or screening purposes, participated in the study, after filling out the informed consent form, the survey questionnaire based survey assessing breast cancer risk factors affecting the breast density and related demographic features, was conducted. SPSS 11.5 software and chi-square, t-test and logistic regression tests were used to analyze the data.

**Results:**

Most of patients (75%) in categories 2 and 3 of mammographic density had a breast density of 51.9%, however, this amount was less (49.2%) in screening mammograms, while in diagnosing group it was more (51.6%). The Findings showed an increase in age, body mass index (BMI), duration of breast feeding, and also to be menopause e, unemployed and married, younger than 29 years old at first delivery, having children up to 8 and smoking are associated with less breast density. Diagnostic mammograms and symptomatic patients showed denser breasts. But density had no association with oral contraceptives pill (OCP) consumption or hormone replacement therapy or calcium and/or vitamin D consumption, age at menarche and menopause, menstruation cycle phase and family history of breast cancer. Age at the first delivery, menopausal status and parity were independently associated with breast density.

**Conclusions:**

Density distribution and risk factors prevalence is different among symptomatic patients and the diagnostic mammograms of the screened persons, hence such information should be considered in the patient managements. In order to consider the effect of marriage and parity on decreasing the breast density, basic consultations should be performed. Smokers and obese women may falsely show low breast density while they may be in high-risk group. In this study no specific phase of menstrual cycle is suggested for mammographic examinations.

## 1. Background

Cancer is one of the leading causes of mortality worldwide (about 13% of all mortalities) (1) and its incidence has globally increased to more than two times during the past 30 years (2). Breast cancer is one of the most common cancers amongst women worldwide (about 23% of all cancers) and it is the first cause of mortality due to cancer among women (3). The annual incidence of breast cancer is steadily increasing and this ascending process has a higher rate in countries with low breast cancer incidence (4). In Iran, breast cancer is the most common cancer among women, and studies have shown that it accounts for 24.4% of all neoplasms (5) with a crude incidence rate of 17.81 (6, 7).

Breast cancer incidence is increasing in Iran. Despite development of diagnostic methods, still many patients are diagnosed at developed stages (8, 9) , Based on the comparative reports, the mean age of Iranian women with breast cancer (with a mean age of 47.1 to 48.8) is at least 10 years younger than the women with the same malignancy in developed countries (8, 10). Thus, the early diagnosis of breast cancer plays an important role in a better prognosis and consequently leaded to a reduced mortality rate. Mammography, as the method of choice for population-based breast cancer screening, aims to detect cancer at asymptomatic stages that the treatment is easier with better outcomes (11). The screening programs in some countries have proved this claim (12).

Various environmental and genetic factors have been approved to affect the susceptibility of breast cancer. According to 2008 WHO report, breast cancer is associated with nulliparity, first delivery at an old age, early menarche and late menopause. Using oral contraception pills (OCP) and Hormone Replacement Therapy (HRT) are also reported to be associated with an increased risk of breast cancer. Family history of breast cancer and high mammography density are two breast cancer risk factors which help to detect high risk women in screening programs (2).

As mentioned before, mammography is the recommended screening method for early detection of breast cancer which has a high potency in detecting suspicious lesions. On the other hands, it helps to identify high risk women by determining mammographic breast density. Breast density higher than 75% is associated with a 4-6 folds increase in breast cancer risk (13, 14).

There are several methods to estimate mammographic density of mammograms including qualitative methods such as Wolfe grade, BIRADS classification, Tabar grade, and six-category classification, as well as quantitative method (computer-assisted threshold method) (15). Qualitative methods are observer-base but these methods do not have the limitations that the quantitative methods do, for instance, the tumor volume assessment and estimation from two-dimensional images is possible, because the observer can reconstruct a three-dimensional image of the breast and densities, out of images taken in two different views, in his mind. On the other hand, many common (16, 17) and uncommon (18, 19) benign and malignant lesions may be presented as dense lesions in mammography images that may be ignored in qualitative methods by the physician when estimating the underlying breast tissue density, but this would not be happened using automated methods. In large or multiple lesion cases, the breast density will be falsely diagnosed.

New methods are being developed to minimize such limitations. Further studies should be done to prove the density, estimated by these methods (15). One the most common qualitative methods used to define the breast density is BIRADS classification which was also used in our study.

## 2. Objectives

We expect a higher breast density in women at higher risk of breast cancer, thus it would be associated with other breast cancer risk factors. Few studies have been performed on mammographic density in Iran (20-22), none of which have been allocated to the epidemiologic distribution of mammographic density. Regarding the difference in prevalence of dense breasts and life style in each population, the determinants of breast density differ in each country. So we decided to assess the distribution of this factor among Iranian population and evaluate its association with other risk factors and factors that may affect breast density based on previous studies.

## 3. Materials and Methods

### 3.1. Study Design and Sampling

This was an analytical cross-sectional study carried out in Tehran, Iran in order to estimate breast density distribution and investigate its determinants in Iranian women. 728 of those who had referred to imaging center of Imam Khomeini Cancer Institute either for diagnosing or screening purposes, were participated in this study, after filling out the informed consent form, the questionnaire based survey, assessing breast cancer risk factors affecting the breast density and related demographic features, was conducted. Inclusion criteria: Accessibility to mammographic report, including mammographic density, reported by one of the three radiologists participating in this study. Exclusion criteria: Individuals with a history of bilateral breast cancer were excluded from the study. Cases with a history of unilateral breast cancer, the mammographic information of the contra lateral breast was included in the analysis. Those who had not filled out the informed consent form or the questionnaire or had no accessible mammographic information were excluded.

### 3.2. Sample Size

Based on the prevalence of dense breasts in literature (23, 24), the minimum estimated sample size, was 380 that was increased to 800, considering the design effect (with the assumption of 2 for DF and a power of 80% at 5% significance level). Sixty three questionnaires were not filled out, that indicate a response rate of around 92%.

### 3.3. Measures

According to literature, different variables which may affect the breast density and breast cancer risk factors were determined and demographic questionnaire was designed. This was a 20-item questionnaire including questions of ages, weight, height, marital status, occupational status, obstetric information (menarche age, menopause age, parity number, age at first delivery, etc.) breast cancer risk factors (personal and family history of breast cancer, OCP consumption, etc.) and other factors seem to affect density (ovarian cycle phase, using calcium, etc.).

During one month, about 60 patients participated in a pilot study and evaluations regarding missed data were conducted. Necessary revisions were taken into account to decrease missed data.

Mammographic density was reported by three expert radiologists using BI-RDAS standards lexicon. According to BIRADS lexicon breast density is classified into four groups: ACR I (almost entirely fat or fibro glandular tissue (FGT) < 25%), ACR II (scattered fibro glandular densities or FGT of 25-50%), ACR III (heterogeneously dense or FGT of 50-75%) and ACR IV (extremely dense or FGT > 75%) (25). Reporters are all colleagues who work in the same center. All mammograms (full-digital two-view ones) were taken with the same technique and read on the same system.

### 3.4. Analysis

More than one observer participated in the study; an assessment of the inter-observer agreement was required to analyze the data. In another study, conducted by the authors in the same center, inter and intra-observers variability in interpreting mammograms was evaluated by three radiologists. Based on the results of this study, inter-observer agreement in reporting density is good (kappa = 0.701). Our results are comparable with another study that recently carried out in Isfahan, Iran (11), showing an extensive agreement among reporters in defining the type of density (Kappa = 0.74). So, it was reasonable to perform one analysis for all samples. This procedure was separately done in both diagnostic and screening groups. Descriptive statistics including frequency, percentage, mean and standard deviations were used to describe the study sample.

The dependent variable (mammographic density) was categorized into two levels: low density breast tissue (breast density of ACR groups 1 and 2) and high density breast tissue (breast density of ACR groups 3 and 4). The independent variables were compared in groups. To compare the continuous data, t-test was used. The association of categorical data and density was analyzed by chi-square and Fisher's exact test. For categorical data another analysis using t-test was performed to compare mean density percentage between the two groups. A P-value of less than 0.05 was considered statistically significant. Both univariate and multiple logistic regression analysis were performed to examine the association between density and independent variables. Data were analyzed using the SPSS statistics software version 11.5. The study has been approved by the ethics committee of Tehran University of Medical Sciences. The written informed assent was taken from all participators.

## 4. Results

### 4.1. The Study Sample

728 women participated in the study amongst 184 (25.3%) had undertaken mammography for diagnostic and 542 (74.7%) for screening purposes. Out of 542 patients, 210 (38.7%) mammograms were done for the first time and 332 (61.3%) for follow up.

The mean age of participants was 48.12 (SD = 8.66) ranging from 19 to 83 years. Participants' age distribution had normal distribution. 57.1% of participants had one of the symptoms including pain, feeling mass or thickness in the breast, bloody or watery discharge, pruritus, erythema or nipple retraction. 37.4% of them experienced the symptoms in left, 28.8% in the right breast and 33.8% have bilaterally pain. 57.8% of the patients in screening group were asymptomatic and 2.2% were in diagnostic group. 94.1% of the study sample were married; 41 (5.6%) had personal history of breast cancer and 170 (24.5%) had positive family history of breast cancer. The demographic features of the samples are shown in Table 1. There was a significant difference between diagnostic and screening groups in some factors including age, breast density, personal history of breast cancer, using HRT and menopausal status. 

**Table 1.** Demographic Feature of the Study

	Total (n=728) Mean ± SD	Screening (n=544) Mean ± SD	Diagnostic Mean ± SD	P OR (95% CI)
**Age (y)**	48.1 ± 8.6	48.6 ± 8.3	46.6 ± 9.4	0.012
**BMI**	27.9 ± 5.4	27.8 ± 4.8	28.3 ± 7.0	0.408
**Density (percent)**	48.9 ± 21.5	47.7 ± 21.4	52.5 ± 21.3	0.009
**Menarche age**	13.5 ± 1.5	13.5 ± 1.5	13.5 ± 1.4	0.594
**Parity number**	2.8 ± 1.8	2.8 ± 1.7	3.0 ± 2.3	0.140
**First birth age**	21.5 ± 4.8)	21.6 ± 4.8	21.1 ± 4.7	0.268
**Menopause age**	47.2 ± 5.6	47.3 ± 5.4	47.1 ± 6.2	0.890
**FH of breast cancer positive**				0.087
First degree	70 ± 10.1	57 ± 10.9	13 ± 7.5	
Second degree	87 ± 12.5	68 ± 13.1	19 ± 11.0	
Both	13 ± 1.9	11 ± 2.1	2 ± 1.2	
Negative	524 ± 75.5	385 ± 73.9	139 ± 80.3	
**FH of other cancers positive**				0.123
First degree	73 ± 11.3	56 ± 11.5	17 ± 10.3	
Second degree	69 ± 10.8	57 ± 11.7	12 ± 7.2	
>1 positive FH	18 ± 3.1	17 ± 3.4	138 ± 83.1	
Negative	513 ± 78.9	375 ± 77.5	1 ± 0.6	
**Personal Hx of breast cancer**				0.046, 2.55 (0.98-6.6)
Positive	41 ± 5.6	36 ± 6.6	5 ± 2.7	
Negative	687 ± 94.4	508 ± 93.4	179 ± 97.3	2.55 (0.98-6.6)
**Marital status**				0.850
Single	41 ± 5.9	30 ± 5.8	11 ± 6.2	
Married	651 ± 94.1	485 ± 94.2	166 ± 93.8	
**Breast feeding yes**				0.510
Complete	379 ± 60.9	285 ± 60.0	94 ± 63.9	
Incomplete	176 ± 28.3	141 ± 29.7	35 ± 23.9	
Never	67 ± 10.8	49 ± 10.3	18 ± 12.2	
**Using OCP yes**				0.895
Already	229 ± 36.9	180 ± 37.3	49 ± 35.5	
Still yes	17 ± 2.7	12 ± 2.5	5 ± 3.6	
Never	375 ± 60.4	291 ± 60.2	84 ± 60.9	
**Using HRT yes**				0.033, 2.70 (1.05-6.98)
Already	23 ± 3.2	22 ± 4.0	1 ± 0.5	
Still yes	20 ± 2.7	16 ± 3.0	4 ± 2.2	
Never	683 ± 94.1	504 ± 93.0	179 ± 97.3	
**Using Ca/Vitamin D**				0.176
Ca	14 ± 1.9	12 ± 2.2	2 ± 1.1	
Both	8 ± 1.1	4 ± 0.7	4 ± 2.2	
None	706 ± 97.0	528 ± 97.1	178 ± 96.7	
**Menopausal status**				0.004, 1.70(1.18-2.44)
Yes	274 ± 37.7	221 ± 40.7	53 ± 28.8	
No	453 ± 62.3	322 ± 60.3	131 ± 71.2	
**Employment status**				0.437
Not employed	479 ± 77.0	367 ± 76.3	112 ± 79.4	
Occupied	111 ± 17.9	86 ± 17.9	25 ± 17.8	
Retired	32 ± 5.1	28 ± 5.8	4 ± 2.8	
**Smoking**				0.962
No	469 ± 72.2	361 ± 72.2	108 ± 72.0	
Yes, passive smoker	161 ± 24.7	123 ± 24.6	38 ± 25.3	
Smoker	20 ± 3.1	16 ± 3.2	4 ± 2.7	
**Radiotherapy Hx**				0.335
Yes	43 ± 6.0	35 ± 6.5	8 ± 4.5	
No	670 ± 94.0	502 ± 93.5	168 ± 95.5	
**Mammographic density**				0.017, 0.66 (0.47-0.93)
Low density ACR I	108 ± 15.3	87 ± 16.5	21 ± 11.8	
ACR II	231 ± 32.8	180 ± 34.3	51 ± 28.6	
High density ACR III	301 ± 42.8	217 ± 41.2	84 ± 47.2	
ACR IV	64 ± 9.1	42 ± 8.0	22 ± 12.4	

### 4.2. Breast Density Distribution Among Participants

The Mean mammographic density of the participants was estimated 48.9%, in the categories four of ACR mammographic density these amounts were 15.3%, 32.8%, 42.8% and 9.1% respectively. Most of the patients (75%) were among categories 2 and 3 of mammographic density and dense breasts (ACR 3 and 4) were 51.9%; however this amount was lessened in screening mammograms (49.2%) and in diagnostic group were even more (51.6%), but statistically significant difference was observed (P = 0.017, OR = 0.66, 95 CI = 0,47-0,93). The mean density in diagnostic and screening group was 52.5% and 47.7% respectively and t-test showed significant difference between them (P = 0.009, T = 2.619).

### 4.3. Factors Affecting Breast Density

Age: the distribution of breast density in different aging groups shows that in younger age groups (15-50 years old) high density breasts were more than low density breasts and in older age groups (51-58 years old) this proportion is reversed and the difference of dense breasts between the two age groups is significant (P < 0.001). On the other hand the results showed that in both screening and diagnostic groups, the mean age is significantly different in both groups of high and low density breasts. Table 2 separately compares different risk factors according to breast density in both diagnostic and screening groups. 

### 4.4. Breast Feeding

The chi-square test showed no significant difference in breast feeding behaviors in two breast density groups, but t-test proved that mean density was significantly higher in those without any history of breast feeding (56.7%) compared to those with a history of two years of breast feeding (48.9%) or any duration of breast feeding (48.4%) (P = 0.007 and 0.003 respectively). T-test in diagnostic group showed no relation (P = 0.071); but in screening group we found this relation (P = 0.020).

### 4.5. Menopausal Status

Results in Table 2 shows there is association between menopausal status and density in both screening and diagnostic groups. Mean density of premenopausal women (53.7%) was also significantly higher (P < 0.001) than menopausal women (41.1%). The same results were driven from t-test in diagnostic (P < 0.001) and screening groups (P < 0.001). 

### 4.6. Marital Status

Chi-square test determined an association between marital status and breast density in the screening but not the diagnostic group. However, t-test proved this relation to exist in both screening (P = 0.001) and diagnostic (P = 0.023) groups.

### 4.7. Parity

Analysis showed there is an association between nulliparity and dense breast tissue in diagnostic groups (P = 0.044) but not in the screening one (P = 0.401). T-test showed there is a reverse relationship between parity number and breast density in both groups (Table 2). According to t-test comparing mean breast density in nullipar women (50.7%) with other women (48.1%), the difference was significant (P < 0.001) and this relationship remains significant in higher parity number cut points,(up to the 8th parity). 

### 4.8. HRT

Cross tab analysis in screening and diagnostic groups, demonstrated that there was no significant association between breast density of those who currently and/or previously received HRT and those without a history of HRT. Indeed all the analyses were repeated only for menopausal women, which introduced the same result.

**Table 2.** Association of Density and Breast Cancer Risk Factors in Diagnostic and Screening Groups

	Screening (n=544) Mean ± SD		P-value OR (95% CI)	Diagnostic (n=184) Mean ± SD		P-value OR (95% CI)
	High Density	Low Densit		High Density	Low Density	
**Age, (y)**	46.41 ± 7.5	50.78 ± 8.4	<0.001^a^, (t=6.252)	44.39 ± 8.3	49.87 ± 10.1	<0.001^a^, (t=3.914)
**BMI**	26.82 ± 4.76	28.86 ± 4.7	<0.001^a^, (t=4.294)	27.62 ± 7.6	29.58 ± 5.6	0.171^a^, (t=1.377)
**Menarche age**	13.53 (1.5)	13.59 (1.4)	0.667^a^, (t=0.431)	13.50 (1.4)	13.96 (4.3)	0.341^a^, (t=0.954)
**Parity number**	2.35 (1.5)	3.19 (1.7)	<0.001^a^, (t=5.766)	2.48 (1.9)	3.86 (2.6)	<0.001^a^, (t=3.937)
**First birth age**	22.67 (5.0)	20.59 (4.4)	<0.001^a^, (t=-4.578)	21.57 (5.1)	20.33 (3.7)	0.156^a^, (t=-1.426)
**Last birth age**						
**Menopause age**	47.30 (4.7)	47.24 (5.8)	0.944^a^, (t=-0.071)	46.04 (6.4)	47.71 (6.3)	0.341^a^, (t=0.960)
**Ovarian cycle length**						
**FH of breast cancer**						0.516^b^
Positive	64 (25.2)	68 (26.3)	0.784^b^	18 (18.0)	15 (22.1)	
Negative	190 (74.8)	191 (73.7)		82 (82.0)	53 (77.9)	
**FH of Other Cancers**			0.632^b^			0.074^b^
Positive	50 (21.7)	58 (23.6)		20 (21.1)	7 (10.4)	
Negative	180 (78.3)	188 (76.4)		75 (78.9)	60 (89.6)	
**Personal Hx of breast cancer**			0.925^b^			0.650^c^
Positive	18 (6.9)	18 (6.7)		4 (3.8)	1 (1.4)	
Negative	241(93.1)	249 (93.3)		102 (96.2)	71 (98.6)	
**Marital status**			0.027^b^, 0.41(0.18-0.92)			0.529^c^
Single	20 (8.0)	9 (3.5)		8 (8.7)	3 (4.4)	
Married	229 (92.0)	250 (96.5)		95 (92.2)	65 (95.6)	
**Nulliparity**			0.401^b^			0.044^b^, 0.23 (0.05-1.08)
Yes	22 (9.4)	18 (7.3)		12 (12.0)	2 (3.1)	
No	212 (90.6)	229 (92.7)		88 (88.0)	63 (96.9)	
**Breast feeding**			0.434^b^			0.622^b^
Yes	202 (88.6)	217 (90.8)		80 (87.0)	44 (89.8)	
Never	26 (11.4)	22 (9.2)		12 13.0)	5 (10.2)	
**Using OCP**			0.680^b^			0.957^b^
Yes	93 (39.1)	140 (59.1)		34 (40.5)	20 (40)	
Never	145 (60.9)	97 (40.9)		50 (59.5)	30 (60)	
**Using HRT**			0.216^b^			0.366^c^
Yes	15 (5.8)M	23 (8.6)		2 (1.9)	3(4.2)	
No	243 (94.2)	244 (91.4)		104 (98.1)	69 (95.8)	
**Using calcium**			0.144^b^			1.000^c^
Yes	5 (1.9)	11 (4.1)		4 (3.8)	2 (2.8)	
No	254 (98.1)	256 (95.9)		102 (96.2)	70 (97.2)	
**Menopausal status**			<0.001^b^, 0.32 (0.22-0.46)			<0.001^b^, 0.29 (0.15-0.57)
Yes	73 (28.2)	147 (55.1)		19 (17.9)	31(43.1)	
No	186 (71.8)	120 (44.9)		87 (82.1)	41 (56.9)	
**Employment status**			0.001^b^, 2.06 (1.34-3.18)			0.017^b^, 3.7 (1.2-11.4)
Not employed	164 (69.5)	197 (82.4)		68 (73.9)	42 (91.3)	
Employed	72 (30.5)	42 (17.6)		24 (26.1)	4 (8.7)	
**Smoking**			0.040^b^, 0.32(0.1-1.0)			0.630^c^
No	181 (97.8)	172 (93.5)		68 (97.1)	40 (95.2)	
Smoker	4 (2.2)	12 (6.5)		2 (2.9)	2 (4.8)	
**Radiotherapy Hx**			0.811^b^			1.000^c^
Yes	16 (6.3)	18 (6.8)		4 (3.9)	3 (4.4)	
No	239 (93.7)	247 (93.2)		99 (96.1)	65 (95.6)	
**Cycle phase**			0.181^b^			0.310^c^
Follicular	64 (52.5)	33 (63.5)		31 (64.6)	5 (45.5)	
Luteal	58 (47.5)	19 (36.5)		17 (35.4)	6 (54.5)	

^a^Derived from *t*-test

^b^Derived from Chi-square

^c^Derived from Fisher Exact test

### 4.9. OCP

As showed in Table 2, analysis presented no association between breast density and current and/or previous use of OCP in each group.

### 4.10. Smoking

In screening group, 51.3% of those who had no exposure to the smoke (non-smokers) and 25% of smokers had dense breasts and their difference was significant (P=0.040), but not in screening group. The Mean breast density among non-smokers was significantly higher (50.2%) than smokers (37.5%) (P = 0.012).

### 4.11. Ovarian Cycle Phase

According to cycle length and LMP (last menstrual period) date and the date of referral for mammographic examination, and considering the regularity of the cycle, menstrual cycle phase at the time of mammography was determined. Those with irregular cycles or incomplete information were not included in the analysis. Neither in screening nor the diagnostic group, had not any density associated to the cycle phase (Table 2). 

### 4.12. Employment Status

In the screening group, the frequency of dense breasts among housewives (45.4%), currently employed women (67.4%) and employed or retired women (63.2%) was compared and significant difference was demonstrated between the mentioned groups (P = 0.016 and P = 0.017 in sequence). in diagnostic group 61.8% of housewives had dense breasts which was significantly lower than dense breasts in currently employed (87.5%, P < 0.001) and retired ones (85.7%, P < 0.001).

### 4.13. BMI

**Figure 1.**
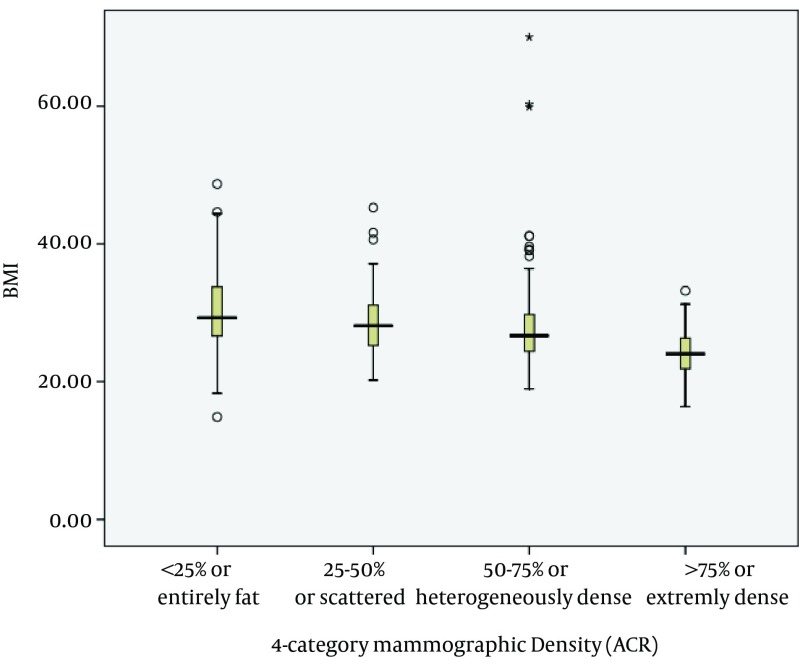
BMI Distribution According to Density

BMI distribution in four ACR density categories is shown (Figure 1). In the screening group the mean BMI in high density breast group (26.8) was significantly less than that individuals with low breast density (28.9) and the difference was significant (P < 0.001). In the diagnostic group the above mentioned association, did not exist.

### 4.14. First Delivery Age

Mean age at first delivery in screening group was 22.7 in high breast density group which was significantly (P < 0.001) higher than low breast density group (20.59) but in diagnostic group this amount was 21.6 and 20.3 years, respectively and the differences were not significant (P = 0.156). Mean breast density was significantly different in women with a first delivery age older and younger than 20 years old (P < 0.001) and this difference was significant up to first delivery age of 29.

### 4.15. Menopause age, Menarche age, Age at Last Delivery and Cycle Length

None of the above mentioned factors showed association with breast density in neither diagnostic nor the screening groups (Table 2). Considering the fact that the association of some of these factors with breast density may affect it through some other ones, binary and multiple logistic regression tests were administered to investigate the interactive effect of these factors on density. Binary logistic regression analysis showed that menopausal status, parity and age at the first delivery were independently associated with breast density (Table 3). Multiple logistic regression test revealed that only menopausal status had association with breast density (P = 0.003).

**Table 3.** Results of Logistic Regression Analysis for Breast Density

	Screening (n=544)		Diagnostic (n=184)		Total (n=728)	
	P-value, Unadjusted OR (95%CI)	P-value, Adjusted OR (95%CI)	P-value, Unadjusted OR (95%CI)	P-value, Adjusted OR (95%CI)	P-value, Unadjusted OR (95%CI)	P-value, Adjusted OR (95%CI)
**Age **	<0.001, 0.93 (0.91-0.95)	0.558, 1.01 (0.97-1.07)	<0.001, 0.93(0.9-0.97)	0.921, 0.997 (0.942-1.056)	<0.001, 0.93 (0.91-0.95)	0.400, 1.02 (0.98-1.07)
**BMI**	<0.001, 0.91 (0.87-0.95)	0.081, 0.95 (0.89-1.00)	0.188, 0.96 (0.91-1.02)	NI^a^	<0.001, 0.93 (0.89-0.96)	0.196, 0.97 (0.93-1.01)
**Parity number**	<0.001, 0.71 (0.63-0.81)	0.011, 0.66 (0.48-0.91)	<0.001, 0.74 (0.63-0.88)	0.167, 0.85 (0.68-1.06)	<0.001, 0.73 (0.66-0.81)	0.025, 0.75 (0.62-0.96)
**First birth age**	<0.001, 1.1 (1.05-1.15)	0.129, 1.06 (0.98-1.15)	0.159, 1.06 (0.98-1.16)	NI	<0.001, 1.09 (1.05-1.13)	0.052, 1.07 (0.99-1.15)
**Employment status**	0.001	0.631	0.023	0.280	<0.001	0.500
Housewife	1 (ref)	1 (ref)	1 (ref)	1 (ref)	1 (ref)	1 (ref)
Employed	2.06 (1.33-3.18)	1.19 (0.59-2.39)	3.71 (1.20-11.43)	1.98 (0.57-6.85)	2.15 (1.45-3.19)	1.24 (0.67-2.30)
**Smoking**	0.050	0.289	0.603	NI	0.025	0.313
Active Smoker	0.56 (0.32-1.00)	0.67 (0.32-1.40)	0.77 (0.28-2.08)	NI	0.55 (0.33-0.93)	0.73 (0.39-1.35
No exposure	1 (ref)	1 (ref)	1 (ref)		1 (ref)	
**Marital status**	0.031	1.000	0.388		0.021	1.000
Married	0.41 (0.18-0.92)	0.000	0.55 (0.14-2.14)	NI	0.44 (0.22-0.88)	0.000
Single	1 (ref)		1 (ref)	NI	1 (ref)	
**Menopausal status**	<0.001	0.001	<0.001	0.116	<0.001	<0.001
Menopaused	0.32 (0.22-0.46)	0.27 (0.12-0.60)	0.29 (0.15-0.57)	0.43 (0.15-1.23)	0.30 (0.22-0.42)	0.27 (0.13-0.54)
Non menopaused	1 (ref)	1 (ref)	1 (ref)	1 (ref)	1 (ref)	1 (ref)
**Mammo. purpose**					0.018	0.033
Screening diagnostic	NA^a^	NA	NA	NA	0.66 (0.47-0.93), 1 (ref)	0.51 (0.27-0.95), 1 (ref)

^a^Abbreviations: NI, Not included in the analysis; NA, Not applicable

## 5. Discussion

Study results showed that the majority of women were among ACR-BIRADS mammographic density categories II and III. The population of women with dense breasts (density>50%) were more than those with low breast density (51.9% versus 48.1%). In diagnostic group the proportion of dense breasts was significantly more than the screening ones.

It seems that Iranian population has denser breasts compared to what has been reported in studies in other countries. Wolf et al. (17) obtained a percentage of 25% dense breasts in the control group versus 37% in the study group. In a case-control study carried out by Saftlas et al. (26), participants were divided into five density categories (< 5%, 5-25%, 25-45%. 45-65% and > 65%) and results revealed that 45% of cases and 32% of controls had mammographic density of 45% and higher (26-28). According to our study results, age, BMI, menopausal status, employment status, marital status, age at the first delivery, breast feeding, parity number and smoking showed association with density. Mammographic purpose and symptomatic or asymptomatic conditions were also related with density (29).

In younger age groups, the percentage of women with high density breasts is more than low density ones. As the age Increases, the breast density decreases is correlated with other studies (16, 20, 23, 26, 30, 31) Maximal breast density was observed in the age group 30-50 years old which correlates with their hormonal pattern. Density distribution differences between screening and diagnostic groups may have been due to some other confounding factors, which would affect density; on the other hand, there are theoretically differences between the two groups. Analysis comparing these factors in screening and diagnostic group revealed a significant difference concerning the age, personal history of breast cancer, using HRT and menopausal status. Among all these factors only age and menopausal status were associated with density which suggests that age and menopausal status may play a role as a confounder on the difference between diagnostic and screening group. Logistic regression test verified this claim. In other words diagnostic group contains dense breasts because it includes younger women and more proportion of premenopausal ones (11, 32, 33).

Unlike parity and menopausal status, age was not independently associated with density. It seems that parity and menopausal status confound the relation of age and density, as claimed by Modugo et al. (30). They have shown by adjusting parity and BMI, no significant association between age and breast density were presented (34).

Unlike Modugo et al. (30) who demonstrated no association between density and breast feeding in Unites States, we found that the breast density was significantly more in those who had never experienced breast feeding and this suggests that breast feeding may change breast cancer risk by changing the breast density. Considering the fact that the mean parity number of women in developing countries is more than that reported in the United States, the above conflict is debatable. Thus, we suggest further studies focused on how duration of breast feeding affects the breast density. By comparing the mean age, no significant difference between those with and without breast feeding was observed (35, 36).

Breast density of premenopausal women was more than menopause group which can be due to the hormonal pattern. Regarding to this fact, we expected to discover an association between the use of HRT/OCP and breast density, as recommended in other studies (20, 24, 31), but we did not. Because of limited number of hormone users in our study, besides the infeasibility of getting exact information about the certain duration of using hormones, the results are not enough reliable and it is necessary to design studies focusing on this association independently.

The breast density was significantly more among nonsmokers. This may be due to age differences; but mean age of smokers and nonsmokers did not show any significant difference (37).

Employed women’s breast density was significantly higher than housewives without a significant age difference. As most of employed women have physical activity with their hands and academic education, and are under stress more than others, this factors may affect this relationship. There are few authors studying such factors. As an example we can name studies conducted in U.S. (17, 26, 38) investigating on education. They proved that smoking and education had a reverse correlation with breast density in menopause women but not in premenopausal ladies (26, 39). According to our study outcomes, smoking seems to have the same effect, but having job associates with an increase in breast density.

Nulliparous women had denser breasts than the others and this difference is significant in woman with higher parity numbers (up to 8). This association can be explained by younger age of nulliparous women and by considering hormonal changes during each pregnancy. Comparing the age difference between these two groups, we found no significant difference (40, 41).

High breast density group showed older age at the first delivery. On the other hand, mean breast density was significantly different in women with the first delivery age older and younger than 20 years old and this difference was significant up to first delivery age of 29. These findings correlate with the effects of first delivery age and breast cancer risk. Other studies (20, 26, 30, 31) reported similar findings. For example, nulliparity (20, 26, 31, 42) and first delivery at old age (31) are proved to be associated with an increase in breast density in menopause and premenopausal women. However some others have denied the association between first delivery age and breast density (17, 43).

Outer study, like other studies (24, 26, 31) showed a reverse association between BMI and density. Breast density of those undergone mammography in follicular phase have not shown any significant difference with those in luteal phase and according to our results no specific time is suggested for mammographic examination. But because of pain in premenstrual period, this time is not recommended for mammography examinations.
